# Phosphorylation of RNF213 by ATM-mediated ubiquitination of RPA1 regulates homologous recombination repair and chemosensitivity

**DOI:** 10.1038/s41419-025-08041-w

**Published:** 2025-10-21

**Authors:** Dingwen Hu, Wenli Wu, Chenhao Wu, Jun Li, Bin Ruan, Heng Cheng, Yanxia Jiang, Yili Wang, Zhiwang Song

**Affiliations:** 1 Department of Medical Care Center, Hainan Women and Children’s Medical Center, Haikou, Hainan China; 2 Department of Oncology, Mayo Clinic, Rochester, MN USA; 3 Department of Endocrinology and Metabolism, the First Affiliated Hospital, Jiangxi Medical College, Nanchang University, Nanchang, Jiangxi China; 4 Prevention and Cure Center of Breast Disease, The Third Hospital of Nanchang City, Nanchang, Jiangxi China; 5 Department of Oncology, Shanghai General Hospital, Shanghai Jiao Tong University, School of Medicine, Shanghai, China; 6 Department of Oncology, the First Affiliated Hospital, Jiangxi Medical College, Nanchang University, Nanchang, Jiangxi China

**Keywords:** Ubiquitylation, Breast cancer

## Abstract

Replication protein A1 (RPA1) is a crucial regulator of homologous recombination (HR) repair and DNA end resection. Studies have demonstrated that the expression and activity of RPA1 are regulated through posttranslational modifications. However, the exact molecular mechanism through which RPA1 activity is regulated remains unclear. Here, we discovered that RNF213 interacts directly with and ubiquitinates RPA1, thereby inhibiting HR repair and DNA end resection. Furthermore, RNF213 is phosphorylated by ATM at Ser217 following DNA damage, which increases the catalytic activity of RNF213. In addition, RNF213 overexpression sensitizes triple-negative breast cancer (TNBC) cells to PARP inhibitor (PARPi) treatment in an RPA1-dependent manner both in vitro and in vivo. Taken together, our findings reveal that RNF213 modulates the response of TNBC cells to PARPi treatment by regulating the ubiquitination of RPA1 and inhibiting HR repair.

## Introduction

Exogenous and endogenous factors that induce DNA damage, including carcinogens, replication stress, and radiation, destroy genome stability and integrity in mammalian cells and result in many human diseases and pathogenic conditions, such as premature aging, immune deficiency, developmental defects, and cancer [1–3]. Mammalian cells have evolved a complex DNA damage response (DDR) system to detect and repair damaged DNA to eliminate the risk of double-strand breaks (DSBs) [4, 5]. Typically, DSBs are mainly repaired through either the homologous recombination (HR) pathway or the nonhomologous end joining (NHEJ) pathway. NHEJ is considered a highly error-prone DNA repair mechanism that directly ligates broken DNA ends without sequence homology and is operational throughout the cell cycle [6]. In contrast, HR is an error-free repair mechanism that is active primarily in the S and G2 phases when intact sister chromatids are available [7].

The choice between HR and NHEJ is determined by whether DNA end resection, which generates 3’ single-stranded DNA (ssDNA), is initiated [8]. DNA resection blocks canonical NHEJ and ensures that the DSB will be repaired via HR [9]. The ssDNA tracks are then quickly covered by the ssDNA-binding protein RPA [10–12], which contributes to termination of end resection [13]. Eventually, RPA is displaced by the strand exchange factor RAD51, a key player in HR [14, 15]. HR deficiency results in genomic instability and cancer predisposition [16–18].

Ubiquitination and deubiquitination are widely accepted as crucial posttranslational modifications (PTMs) that play essential roles in controlling the activity and stability of key proteins and subsequently regulating numerous physiological and pathological processes [19–21]. Numerous studies have shown that the ubiquitination machinery also governs the DDR and helps to maintain genome stability [22, 23]. For example, USP52 interacts with and deubiquitinates CtIP to facilitate the phosphorylation of CtIP at Thr847 and its consequent activation [24]. USP13 binds to and stabilizes TopBP1 through deubiquitination and orchestrates the replication stress response [25]. In addition, RNF168/RNF18-mediated ubiquitination plays a crucial role in recruiting key repair factors such as 53BP1 and RAP80 to DSB sites [26, 27]. RNF19A disrupts the BRCA1**–**BARD1 complex by ubiquitinating BARD1 and inhibits HR [28]. However, the ubiquitination status of replication protein A1 (RPA1) has not been fully elucidated.

In this study, we show that RNF213, a member of the ring-between-ring fingers (RBR) family of E3 ligases, binds to and ubiquitinates RPA1. Additionally, RNF213 inhibits DNA end resection and HR following DNA damage and sensitizes triple-negative breast cancer (TNBC) cells to PARP inhibitor (PARPi) treatment both in vitro and in vivo. Moreover, our clinical analysis revealed that RNF213 expression is significantly associated with better prognosis in TNBC patients. In conclusion, our results demonstrate the role and mechanism of RNF213 in regulating the stability and activity of RPA1 and reveal novel potential targets for cancer treatment.

## Materials and methods

### Quantification of DNA resection

ER-AsiSI U2OS cells expressing the indicated short hairpin RNAs (shRNAs), small interfering RNAs (siRNAs), or constructs were harvested after treatment with 1 μM 4-hydroxytamoxifen (4-OHT) for 4 h. DNAzol (Invitrogen) was used to extract genomic DNA according to the manufacturer’s instructions. Then, 500 ng of genomic DNA was incubated with a mock control or digested with the BsrGI restriction enzyme at 37 °C overnight. Two microliters of DNA was used as the template in a 25 μl qPCR mixture containing 12.5 μl of 2× TaqMan Universal PCR Master Mix-ABI, each primer (0.5 μM) and the probe (0.2 μM). We calculated the ΔCt value by subtracting the Ct value of the mock-digested sample from the Ct value of the digested sample. Finally, the percentage of ssDNA was calculated with the following equation: ssDNA% = 1/(2(ΔCt−1) + 0.5) × 100.

### RNF213-deficient mice

All animal experiments were performed in accordance with the National Institutes of Health Guide for the Care and Use of Laboratory Animals, and the protocols were approved by the Institutional Animal Care and Use Committee. RNF213-deficient mice on a C57BL/6 background were purchased from the Laboratory Animal Center. The animals were maintained in a specific pathogen-free (SPF) animal care facility.

### Xenograft model and TNBC patient-derived xenografts (PDXs)

Control or Flag-RNF19A MDA-MB-231 cells were injected subcutaneously into the flanks of 6-week-old female athymic BALB/c nude mice (National Cancer Institute/National Institutes of Health) to establish mouse xenograft models. Each mouse received injections of 100 μl of a mixture of 2 × 10^6^ cells in PBS with growth factor-reduced Matrigel (2:1 ratio; BD Biosciences). PDX models were established in 5-week-old female BALB/c nude mice. First, we implanted fresh human TNBC tumor fragments into the flanks of the mice. We then monitored the health of the mice and the growth of the tumors, collected the tumors when the diameter reached 2 cm, and reimplanted the tumors into new mice. Mice bearing tumors with a volume of ~100 mm^3^ were randomly allocated to the vehicle (saline) or olaparib intervention (50 mg/kg/day) groups. The tumors were measured every 3 days with calipers beginning at treatment initiation, and the tumor volume was calculated with the formula length × width^2^. After 3 weeks of treatment, all the mice were sacrificed, and the tumors were harvested.

### Immunohistochemical (IHC) staining

A total of 86 formalin-fixed, paraffin-embedded TNBC tissues and paired normal tissue samples, along with the corresponding clinicopathological data, were collected at Hainan Women and Children’s Medical Center. IHC assays were performed according to the standard streptavidin‒biotin labeling protocol (Dako, Carpinteria, CA, USA). A semiquantitative scoring system developed in Germany was utilized to assess the expression of RNF213 on the basis of the staining area and intensity [29].

The expression level of RNF213 in both the tumor tissues and the adjacent normal tissues was classified as high (score ≥4) or low (score <4). The immunostaining scores were determined by two independent pathologists in a blinded manner to avoid potential bias. All these studies were carried out with informed consent from all the subjects and were approved by the Medical Ethics Committee.

### Statistics

For the in vitro studies, the data are presented as the mean ± SEM of at least three independent experiments. The animal study data are presented as the mean ± SEM of the data from five mice. Statistical analyses were performed with SPSS Statistics and GraphPad Prism 7.0, and two-tailed Student’s t test and analysis of variance (ANOVA) were used to compare data between two groups and among more than two groups, respectively. The Kaplan‒Meier method and the log–rank test were adopted to analyze overall survival. * indicates *P* < 0.05, ** indicates *P* < 0.01, *** indicates *P* < 0.001, and n.s. (not significant) indicates *P* > 0.05.

## Results

### RNF213 interacts with and ubiquitinates RPA1

**Fig. 1:**
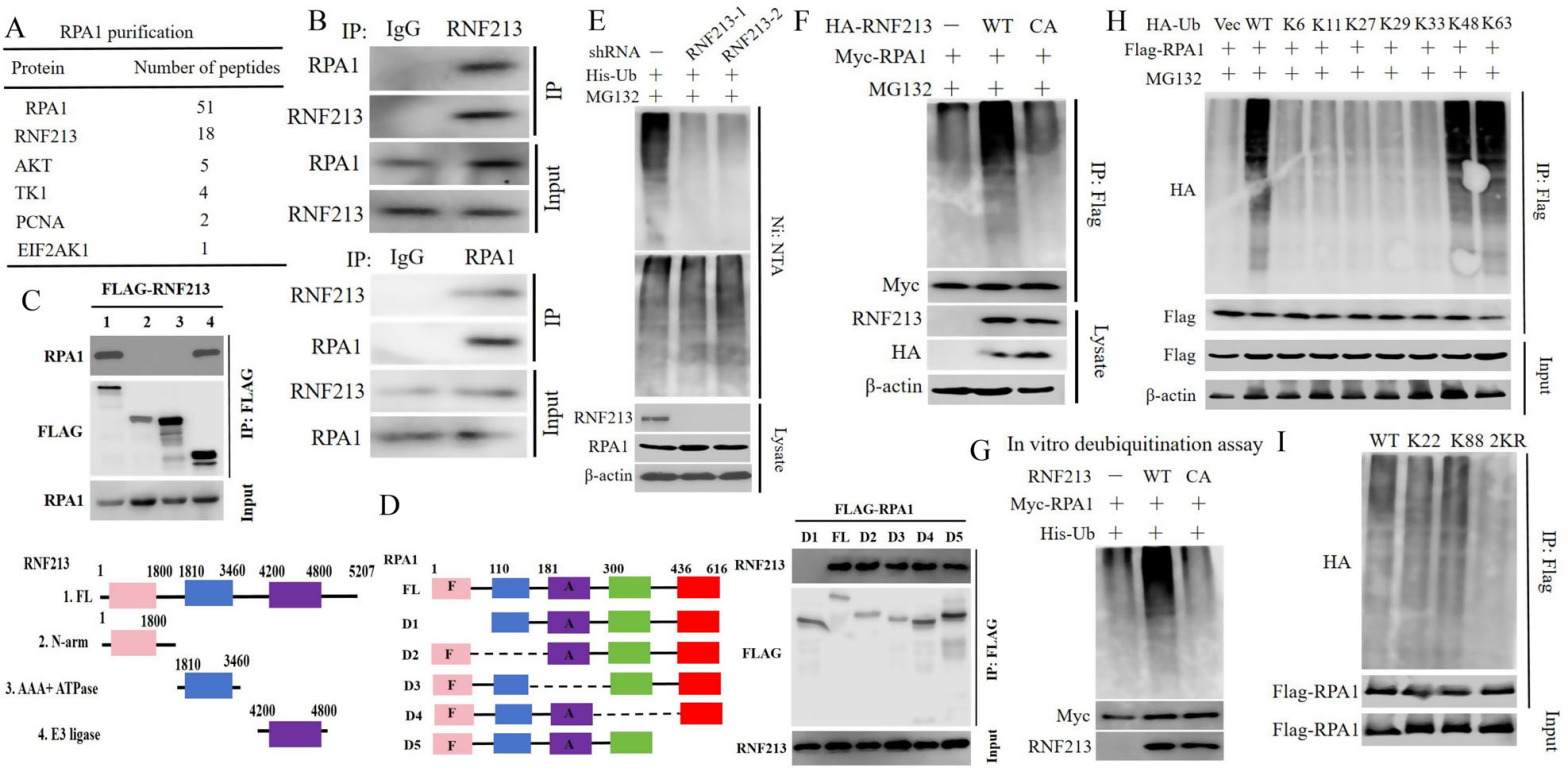
RNF213 interacts with and ubiquitinates RPA1. **A** List of RPA1-associated proteins identified by mass spectrometric analysis. HEK293T cells stably expressing Flag-RPA1 were generated and RPA1 complexes were subjected to mass spectrometric analysis. **B** HEK293T cell lysates were subject to immunoprecipitation with control IgG, anti-RNF213 or RPA1 antibodies. The immunoprecipitates were then blotted with indicated antibodies. **C** HEK293T cells were transiently transfected with the indicated RNF213 constructs for 24 h, then cell lysates were incubated with anti-Flag agarose beads overnight at 4 °C. The immunoprecipitates were then blotted with indicated antibodies. **D** HEK293T cells were transiently transfected with the indicated RPA1 constructs for 24 h, then cell lysates were incubated with anti-Flag agarose beads overnight at 4 °C. The immunoprecipitates were then blotted with indicated antibodies. **E** HEK293T cells stably expressing control (Ctrl) or RNF213 shRNAs were transfected with indicated constructs. After 48 h, cells were treated with MG132 for 6 h before collecting and Ni-NTA beads were used to pull down His-tagged ubiquitin. Blots were probed with the indicated antibodies. **F** HEK293T cells stably expressing wild-type RNF213 (WT) or the catalytically inactive mutant (RNF213 CA) were transfected with Myc-RPA1. After 48 h, cells were treated with MG132 (10 μm) for 6 h before collecting. Cell lysates were immunoprecipitated with Myc-tagged beads. The polyubiquitylated RPA1 protein was detected by anti-ubiquitin antibody. **G** Ubiquitination of RPA1 in vitro by RNF213. Ubiquitinated RPA1 was incubated with purified RNF213 WT or RNF213 CA in vitro, and then blotted with the indicated antibodies. **H** HEK293T cells with RNF213 shRNAs were transiently transfected with Flag- tagged RPA1 and various Ub mutant plasmids. After 48 h, cells were treated with MG132 (50 μM) for 3 h, and a ubiquitination assay was performed. Cell lysates were subjected to immunoprecipitation with anti-Flag agarose beads, and then blotted with the indicated antibodies. **I** HEK293T cells were transfected with His-Ub and the indicated RPA1 constructs with different mutated ubiquitination sites for 24 h. Cell lysates were incubated with nickel (His) beads and then blots were probed with indicated antibodies.

RPA1 plays a crucial role in DNA end resection and DNA DSB repair [30]. Previous studies have shown that several E3 ligases or their substrate adapters control RPA1 ubiquitination and protein stability. However, the process of RPA1 ubiquitination remains unclear. Tandem affinity purification and mass spectrometry analysis were performed, and several proteins potentially interacting with RPA1 were identified (Fig. 1A). Next, a coimmunoprecipitation (co-IP) assay was performed to confirm the interaction between endogenous RNF213 and RPA1, and the results revealed that RPA1 was coimmunoprecipitated with RNF213 in HEK293T cells; reciprocal IP with an anti-RNF213 antibody also resulted in the pulldown of RPA1 (Fig. 1B). In addition, we mapped the binding region(s) between RNF213 and RPA1 via several truncation mutants of RNF213 and RPA1. As shown in Fig. 1C, D, the E3 ligase domain of RNF213 and the N-terminal region (amino acids 1**–**100) of RPA1 are indispensable for the interaction between RNF213 and RPA1.

Moreover, RNF213 deficiency impaired the ubiquitination of RPA1, whereas compared with overexpression of the CA mutant (RING mutant), overexpression of wild-type RNF213 (RNF213-WT) increased RPA1 ubiquitination (Fig. 1E, F). Moreover, the overexpression of HA-tagged RNF213 increased the RPA1 ubiquitination level in a dose-dependent manner (Supplementary Fig. 1A). Next, an in vitro deubiquitination assay was conducted to further confirm whether RNF213 directly ubiquitinates RPA1, and we found that recombinant RNF213-WT but not the RNF213 CA mutant (RNF213-CA) ubiquitinated RPA1 in vitro (Fig. 1G). There are 7 main lysine (K) residues, namely, K6, K11, K27, K29, K33, K48 and K63, to which ubiquitin chains are conjugated, and these linkage types have different cellular consequences [31, 32]. We sought to identify the types of ubiquitin linkages and observed that RPA1 was extensively populated with K48-linked (Ub-K48) and K63-linked (Ub-K63) ubiquitin chains (Fig. 1H). We further determined the statuses of these linkages (Ub-K48 and UbK63) with and without RNF213 overexpression and found that RNF213 overexpression increased only the K48-linked polyubiquitination of RPA1 (Supplementary Fig. 1B). Furthermore, the overexpression of RNF213-WT but not RNF213-CA increased the K48-linked polyubiquitination of RPA1 (Supplementary Fig. 1C). We then mapped the potential sites of RPA1 whose ubiquitination is regulated by RNF213. We found that K22 and K88, located within the N-terminus of RPA1, were indispensable for the interaction of RPA1 with RNF213. Therefore, we mutated each of these ubiquitination sites in RPA1 (i.e., KR mutations) and carried out ubiquitination assays. The double K-to-R (2KR) mutation almost completely abolished the ubiquitination of RPA1 (Fig. 1I). These results reveal that both lysine residues are crucial ubiquitination sites in RPA1.

### RNF213 inhibits HR and DNA end resection

**Fig. 2:**
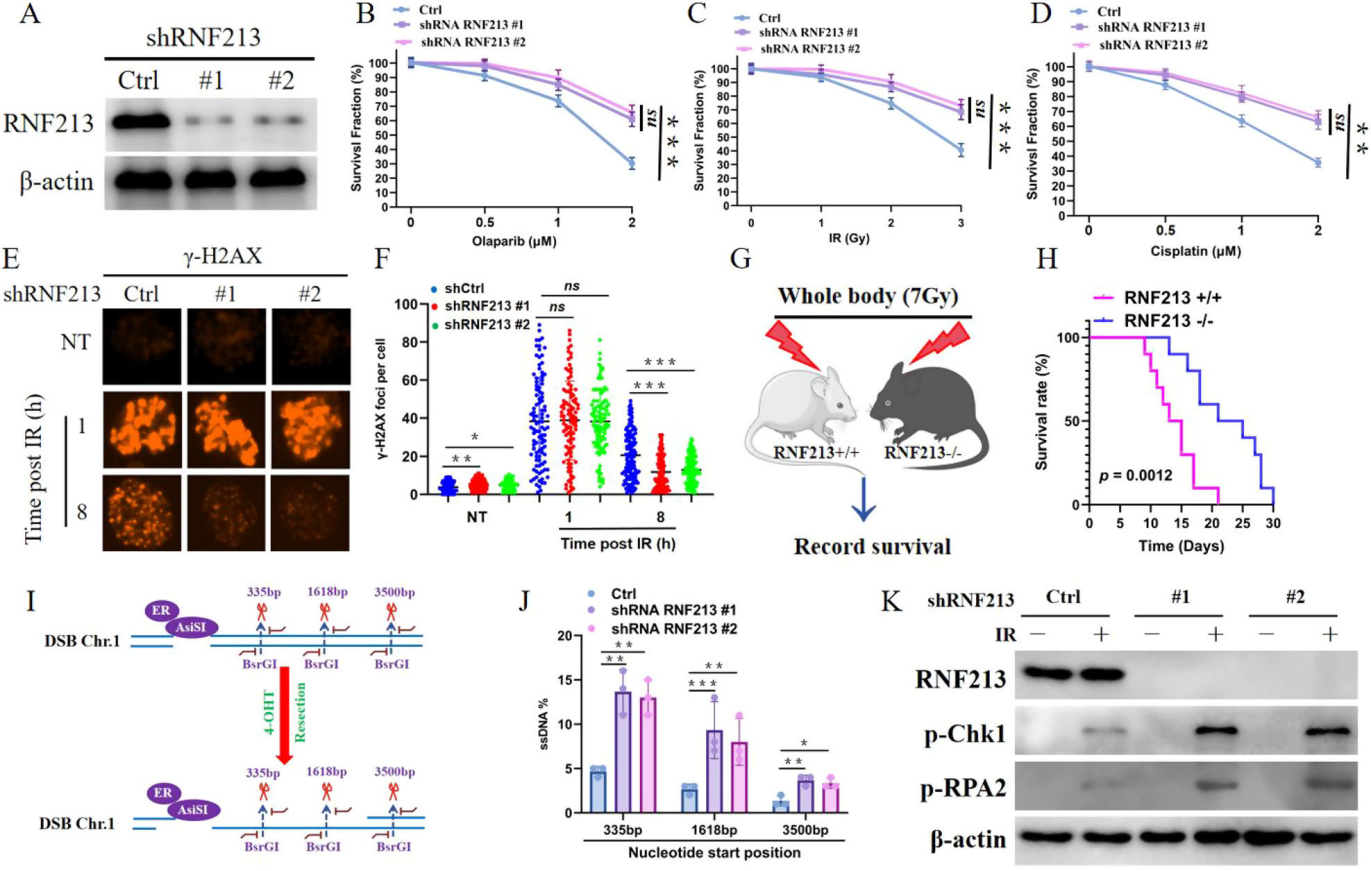
RNF213 inhibits HR and increases sensitivity to DNA-damaging agents. **A** U2OS cells stably expressing Control or RNF213 shRNA, the blots were probed with indicated antibodies. The sensitivity of control (Ctrl) and RNF213 knockdown U2OS cells to Olaparib (**B**), IR (**C**) and cisplatin (**D**) was assessed by colony formation assay. Error bars are means ± s.d. of three independent experiments. **E**, **F** Control and RNF213 knockdown U2OS cells were treated with or without IR (4 Gy), γ-H2AX foci before or 1 h, and 8 h after IR was detected by immunofluorescence. Nuclei were visualized with DAPI (blue). Representative images are shown in (**E**). Quantification of focus signals per cell (each dot represents a single cell, n = 100) is shown in F. Error bars represent means ± s.d. of three independent experiments. **G**, **H** Wild-type (+/+, n = 10), and RNF213 knockout (−/−, n = 10) BALB/C mice were exposed to whole body irradiation (7 Gy) (**G**) and survival was assessed. Comparisons between groups were made using the Log-rank test (**H**). **I** Schematic of ER-AsiSI system for quantification of DNA resection. Restriction enzyme AsiSI is fused to the estrogen receptor (ER) and can be induced to the nucleus and generate DSBs at sequence-specific sites by 4-OHT. The genomic DNA was extracted and quantification of ssDNA generated by resection was measured by qPCR. The primer pairs for DSBs are across BsrGI restriction sites. **J** Control and RNF213 knockdown ER-AsiSI U2OS cells were pretreated with 300 nM 4-OHT for 4 h to induce DSBs. Genomic DNA was extracted and digested or mock digested with BsrGI overnight. DNA-end resection adjacent to indicated sites was measured by qPCR. **K** Control and RNF213-depleted U2OS cells were exposed to IR for 4 h. Cells were then harvested and blotted with indicated antibodies.

Considering the critical role of RPA1 in DNA end resection and HR, we hypothesized that RNF213 might participate in the regulation of the DDR. First, we sought to determine whether RNF213 is involved in the DDR. We found that RNF213-deficient U2OS cells presented increased resistance to DNA-damaging agents, including olaparib, ionizing radiation (IR), and cisplatin (Fig. 2A–D), but ectopic expression of RNF213-WT reversed this effect (Supplementary Fig. 2A–D). Additionally, RNF213 depletion reduced the accumulation of γH2AX foci at a late time point (8 h) (Fig. 2E, F). Moreover, RNF213 knockout mice were used to further investigate the effect of RNF213 on DNA damage sensitivity in vivo. RNF213 knockout mice were profoundly radioresistant (Fig. 2G, H). Next, a dual-reporter assay for HR and NHEJ was employed to determine whether RNF213 regulates the DDR. RNF213 deficiency increased HR efficiency but did not significantly affect NHEJ efficiency; meanwhile, RNF213 deficiency enhanced alternative end joining (alt-EJ) (Supplementary Fig. 2E). These findings are consistent with previous reports [33]. Importantly, RNF213 deficiency did not significantly change the cell cycle profile (Supplementary Fig. 2F), demonstrating that the change in HR efficiency caused by RNF213 was not due to cell cycle alteration. In addition, RNF213 deficiency increased the number of RDA51 and RPA2 foci but did not affect the number of 53BP1, BRCA1, or CtIP foci (Supplementary Fig. 2G–H). These results confirmed that RNF213 promotes HR repair. The ER-ASISI system was subsequently adopted to examine the resection efficiency. In this system, the nuclear translocation of the restriction enzyme AsiSI is induced via 4-OHT treatment, and AsiSI subsequently generates DSBs at sequence-specific sites (Fig. 2I). RNF213 depletion markedly increased the generation of ssDNA at all distances from the DSB sites (Fig. 2J). The RPA**–**ssDNA complex is crucial for the phosphorylation of CHK1 and RPA to regulate cellular sensitivity to DNA-damaging agents [34]. We found, as expected, that RNF213 depletion increased IR-induced CHK1 and RPA2 phosphorylation (Fig. 2K). Additionally, RPA is well known to be involved in DNA replication in addition to the DNA double-strand break response and repair (DSBRR), and DNA replication occurs only in the S phase [33, 35]. We sought to determine whether RNF213 regulates RPA1 ubiquitination in a cell cycle-dependent manner and found that, as we anticipated, RNF213 predominantly interacted with RPA1 in S-phase cells (Supplementary Fig. 2I). Moreover, in agreement with these observations, RPA1 was highly polyubiquitinated in S-phase cells (Supplementary Fig. 2J). Taken together, these findings show that RNF213 is essential for DSB-induced HR repair and DNA end resection.

### RNF213 regulates HR repair and DNA end resection in a catalytic activity- and RPA1-dependent manner

**Fig. 3:**
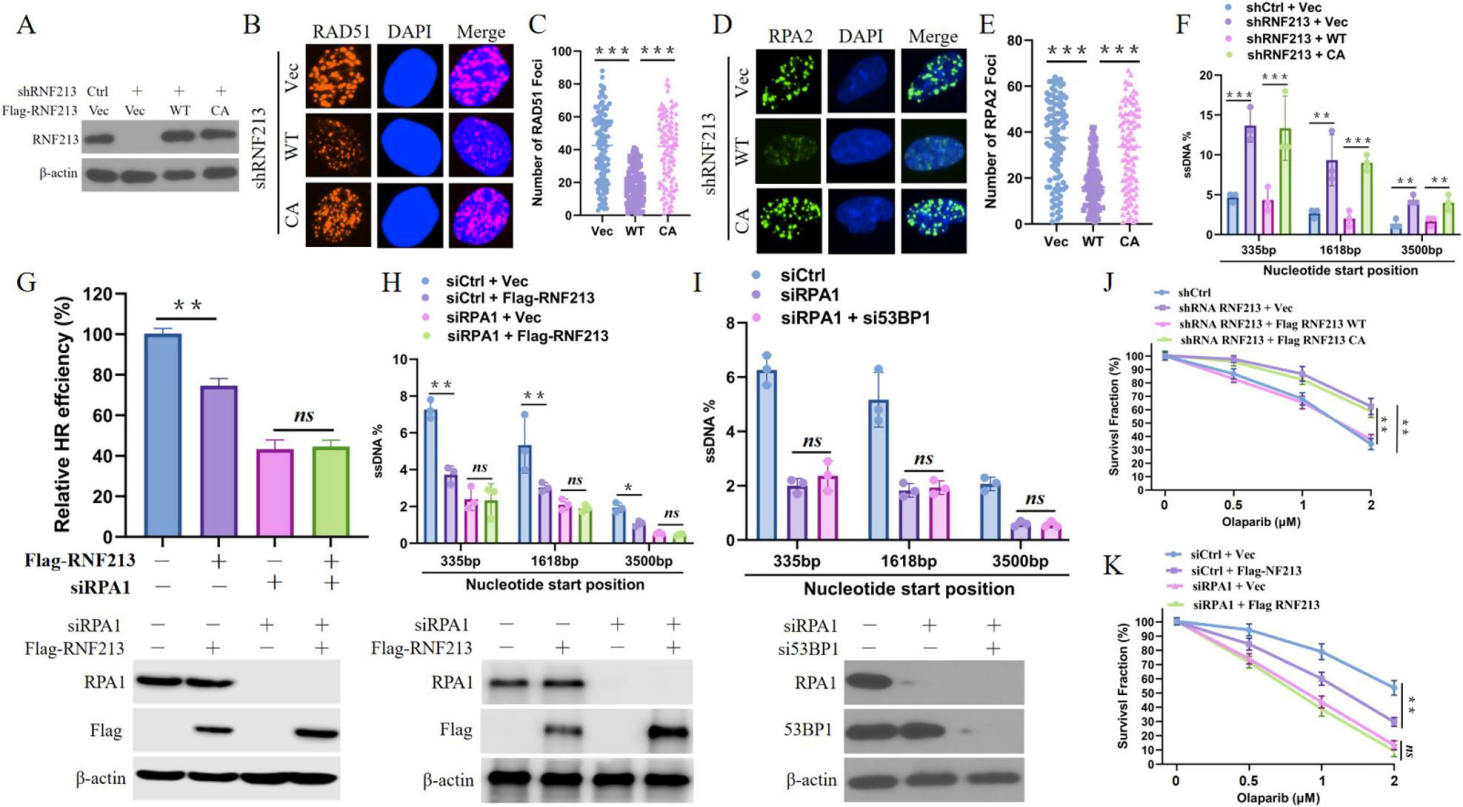
RNF213 functions in end resection through its catalytic activity and in a RPA1-dependent manner. **A** Control and RNF213 knockdown U2OS cells were reconstituted with Vec, WT, or CA Flag-RNF213 and the blots were probed with indicated antibodies. **B**–**E** RNF213 knockdown U2OS cells stably expressing Vec, WT, or CA Flag-RNF213 were treated with IR (3 Gy, 4 h for RAD51 and RPA32). Representative images are shown in (**B**, **D**). Quantification of focus signals per cell (each dot represents a single cell, n = 100) is shown in (**C**, **E**). **F** RNF213 knockdown ER-AsiSI U2OS cells were reconstituted with Vec, WT, or CA Flag-RNF213 and were pretreated with 300 nM 4-OHT for 4 h to induce DSBs. Genomic DNA was extracted and digested or mock digested with BsrGI overnight. DNA-end resection adjacent to indicated sites was measured by qPCR. **G** HEK293T cells stably expressing Vec or Flag-RNF213 were transfected with control (Ctrl) or RPA1 siRNAs for 48 h. HR efficiency was determined using the HR reporter assay. **H** U2OS ER-AsiSI cells stably expressing Vec or Flag-RNF213 transfected with control or RPA1 siRNAs for 48 h and were pretreated with 300 nM 4-OHT for 4 h before digest and measurement of DNA resection. **I** U2OS ER-AsiSI cells transfected with control, RPA1 or/and 53BP1 siRNAs for 48 h and were pretreated with 300 nM 4-OHT for 4 h before digest and measurement of DNA resection. **J** RNF213 knockdown U2OS cells stably expressing Vec, WT, or CA Flag-RNF213 were subjected to colony formation assay for assessment of response to Olaparib. **K** U2OS cells stably expressing Vec or Flag-RNF213 were transfected with control (Ctrl) or RPA1 siRNAs were subjected to colony formation assay for assessment of response to Olaparib.

RNF213-deficient cells reconstituted with RNF213-WT or RNF213-CA were used to further explore whether the effects of RNF213 on HR and DNA end resection are dependent on its catalytic activity. As shown in Fig. 3A–F, RNF213-WT but not RNF213-CA strongly inhibited the formation of RAD51 and RPA32 foci and the generation of ssDNA. These results demonstrated that the catalytic activity of RNF213 is crucial for its effects on HR and DNA end resection. We then investigated whether RNF213 regulates HR and DNA end resection through RPA1. As shown in Fig. 3G–I, overexpression of RNF213 noticeably inhibited HR and ssDNA generation but did not further reduce HR repair or ssDNA generation after the silencing of RPA1. These findings reveal that RNF213 regulates HR and DNA end resection in an RPA1-dependent manner. In addition, RNF213 knockdown did not affect the PARPi sensitivity of RNF213-CA-expressing cells or RPA1-deficient cells (Fig. 3J, K). Taken together, these findings indicate that RNF213 regulates HR repair in a catalytic activity- and RPA1-dependent manner.

### Ubiquitination of RPA1 by RNF213 is important for HR and DNA end resection

**Fig. 4:**
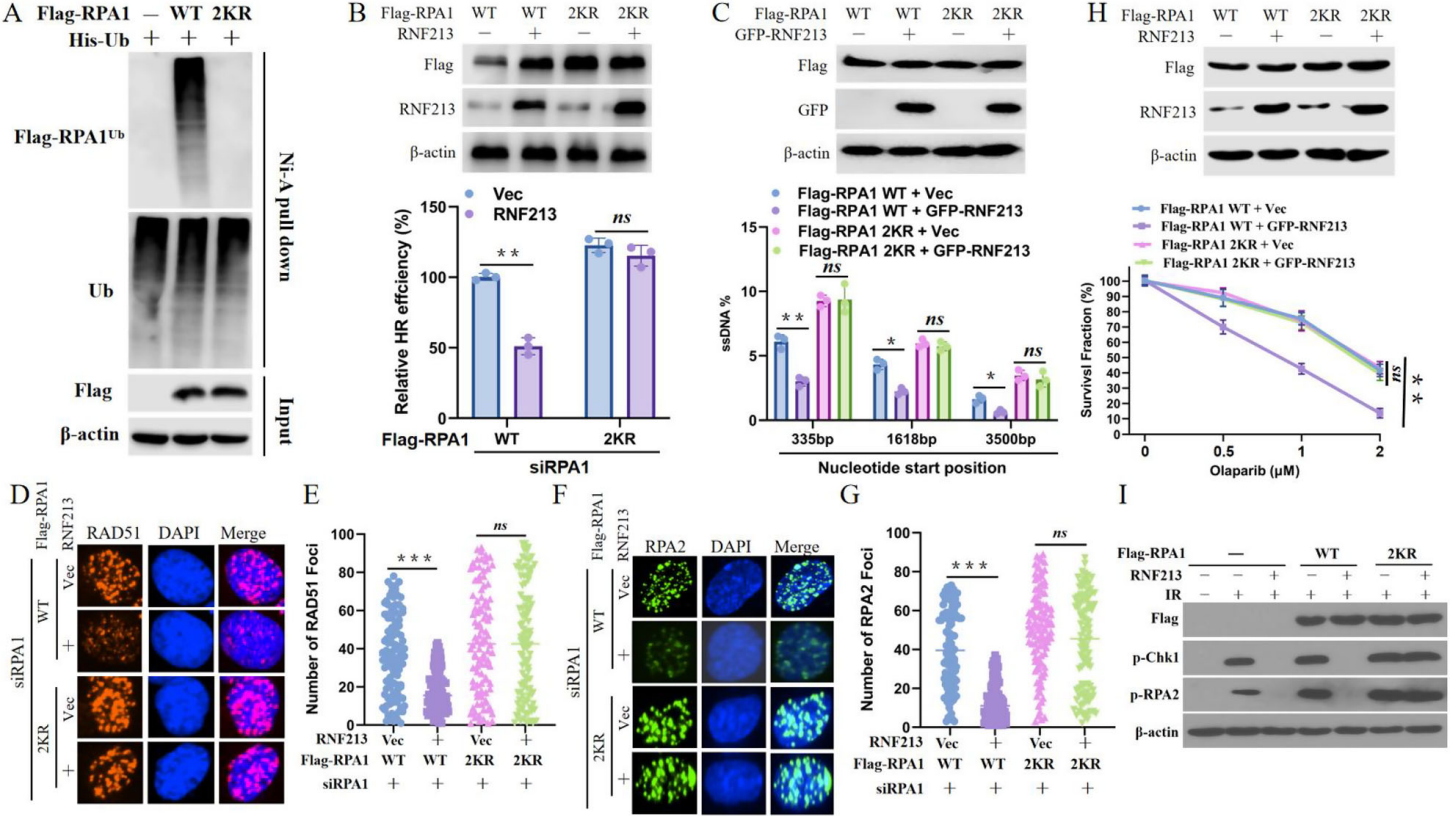
Ubiquitination of RPA1 by RNF213 is important for HR and DNA end resection. **A** HEK293T cells were transfected with WT RPA1 or RPA1 2KR for 24 h before harvesting and immunoprecipitation with nickel (His) beads. Blots were detected by indicated antibodies. **B** The HR-mediated DSB repair efficiency of control or RNF213 over-expression HEK293T cells expressing the indicated RPA1constructs were analyzed using HR reporter. Error bars represent means ± s.d. of three independent experiments. **C** Control or RNF213 over-expression ERAsiSI U2OS cells were transfected with WT RPA1 or RPA1 2KR for 24 h, and then cells were treated with 4-OHT to induce DSB. Cells were harvested for DNA end resection analysis measured by qPCR assay. Error bars represent means ± s.d. of three independent experiments. Representative images (**D**, **F**) and quantification (**E**, **G**) of RAD551 (**D**, **E**) RPA2 (**F**, **G**) in control or RNF213 over-expression U2OS cells which were transfected with WT RPA1 or RPA1 2KR for 24 h before treated with 3 Gy IR for another 4 h. Error bars represent means ± s.d. of three independent experiments. **H** Control or RNF213 over-expression U2OS cells were transfected with WT RPA1 or RPA1 2KR were subjected to colony formation assay for assessment of response to Olaparib. **I** Control or RNF213 over-expression U2OS cells were transfected with WT RPA1 or RPA1 2KR for 24 h before treated with IR. Blots were probed with the indicated antibodies.

As shown in Fig. 1I, mutation of either of the two ubiquitination sites (K22 or K88) in RPA1 noticeably reduced its ubiquitination level. These two sites were mutated simultaneously, and the 2KR mutation almost completely abolished the ubiquitination of RPA1 (Fig. 4A).

Accordingly, we further examined whether RPA1 ubiquitination regulates HR and DNA end resection. As shown in Fig. 4B–G, the expression of RPA1-2KR, but not RPA1-WT, reversed the RNF213 deficiency-induced promotion of HR repair, ssDNA production, and RAD51/RPA2 focus formation. Moreover, we explored the biological effects of RPA1 ubiquitination on the DDR. The expression of RPA1-2KR but not the expression of RPA1-WT reversed the PARPi sensitivity resulting from RNF213 overexpression (Fig. 4H). In addition, the expression of the RPA1 2KR mutant but not the expression of RPA1-WT restored the RNF213 overexpression-induced phosphorylation of CHK1 and RPA2 (Fig. 4I). Collectively, these findings indicate that RNF213 promotes the ubiquitination of RPA1 to inhibit HR repair and DNA end resection following DNA damage.

### Phosphorylation of RNF213 by ATM upon IR exposure

**Fig. 5:**
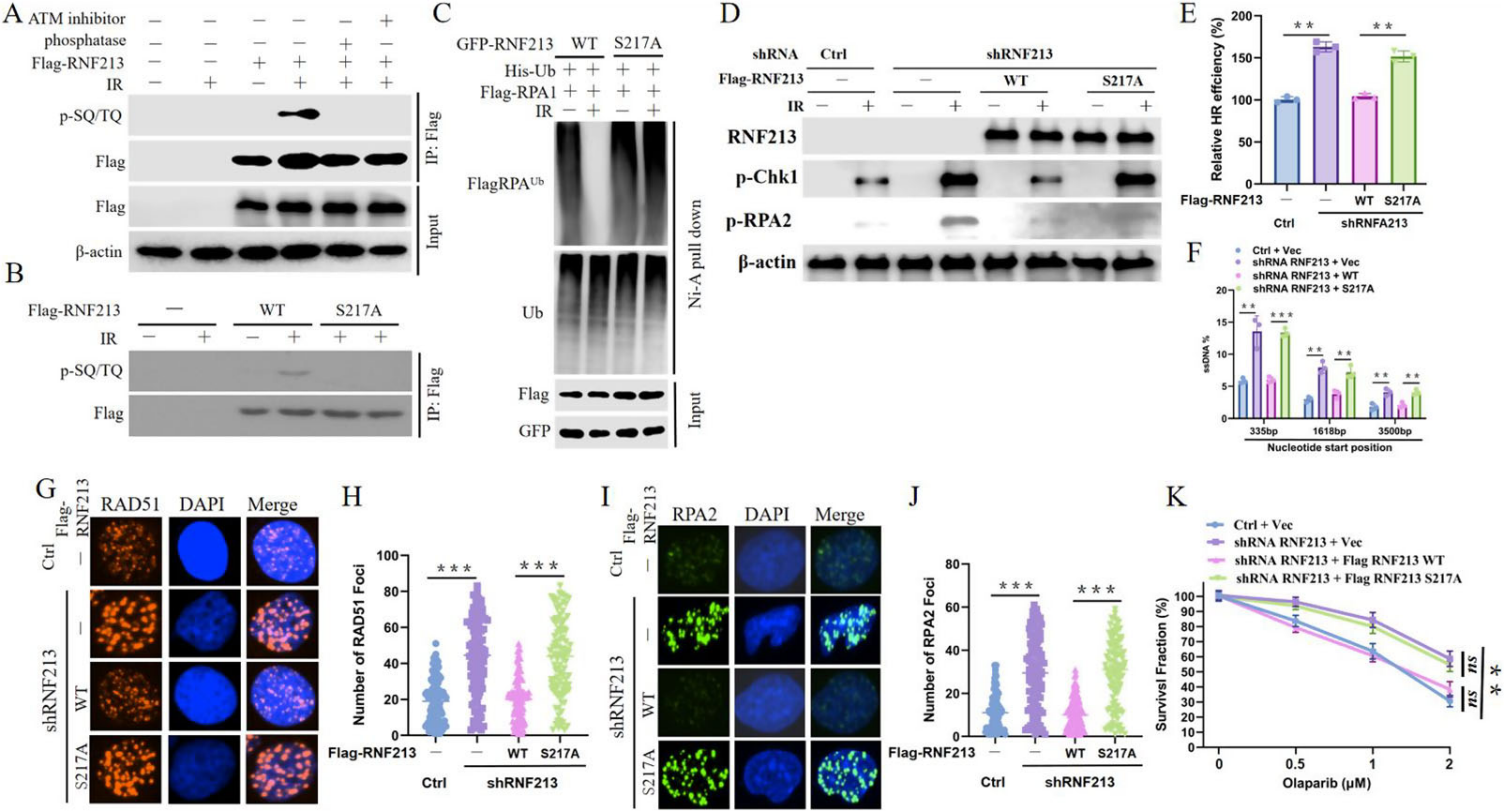
ATM kinase promotes the activity of RNF213 to regulate the DDR. **A** HEK293T cells transfected with Flag-RNF213 were treated with DMSO or 25 μM Ku55933 for 2 h prior to IR treatment. Harvested cells were immunoprecipitated with anti-Flag agarose beads. After untreated or treated with lambda protein phosphatase, blots were probed with pSQ/TQ antibody. **B** HEK293T cells transfected with indicated RNF213 constructs were harvested after IR and then immunoprecipitated with anti-Flag agarose, blots were probed with indicated antibodies. **C** Ubiquitinated RPA1 was incubated with purified Flag-WT RNF213 or the S217A mutant before or after IR to perform deubiquitination reaction assay in vitro, and then blotted with the indicated antibodies. **D** Control or RNF213-depleted U2OS cells were transfected with WT RNF213 or the S217A mutant 24 h before IR. After 2 h, cells were harvested and subjected to blot with the indicated antibodies. **E** Control or RNF213-depleted HEK293T cells transfected with indicated RNF213 constructs together with HR reporter were harvested for HR assay. Error bars represent means ± s.d. of three independent experiments. **F** Control or RNF213-depleted ERAsiSI U2OS cells were indicated RNF213 constructs for 24 h, and then cells were treated with 4-OHT to induce DSB. Cells were harvested for DNA end resection analysis measured by qPCR assay. Error bars represent means ± s.d. of three independent experiments. Representative images (**G**, **I**) and quantification (**H**, **J**) of RAD551 (**G**, **H**) RPA2 (**I**, **J**) in control or RNF213-depleted U2OS cells which were transfected with indicated RNF213 constructs for 24 h before treated with 3 Gy IR for another 4 h. Error bars represent means ± s.d. of three independent experiments. **K** Control or RNF213-depleted U2OS cells transfected with indicated RNF213 constructs were subjected to colony formation assay for assessment of response to Olaparib.

Considering that the ubiquitination of RPA1 is induced by RNF213, we further explored whether the expression of RNF213 itself is mediated by DNA damage repair signaling. Analysis of online data revealed that RNF213 has one Ser-Gln/Thr-Gln (pSQ/TQ) motif (S217), implying that RNF213 is a potential ATM/ATR substrate. In fact, RNF213 can be phosphorylated at SQ/TQ motifs in response to DNA damage induced by IR, and this phosphorylation was abolished by treatment with an ATM inhibitor or treatment of cell lysates with phosphatase (Fig. 5A). Additionally, the S217A mutation almost completely abolished the phosphorylation of RNF213 following IR treatment (Fig. 5B). These data prove that RNF213 is phosphorylated at Ser217 in an ATM-dependent manner following IR treatment. Next, we further examined the biological importance of RNF213 phosphorylation. First, RPA1 ubiquitination in cells overexpressing RNF213-WT was significantly decreased after IR treatment, but RPA1 ubiquitination in cells overexpressing the RNF213-S217A mutant was unchanged (Fig. 5C). These results indicate that RNF213 phosphorylation at S217 plays an important role in regulating the activity of RNF213 deubiquitinases (DUBs); thus, we investigated the regulatory effects of RNF213 phosphorylation on HR and DNA end resection. The expression of RNF-S217A but not RNF213-WT reversed the RNF213 depletion-induced increase in CHK1 and RPA2 phosphorylation (Fig. 5D), increased HR efficiency (Fig. 5E) and ssDNA production (Fig. 5F), and reduced RAD51/RPA2 focus formation (Fig. 5G–J) and PARPi resistance (Fig. 5K). Collectively, these results indicate that RNF213 phosphorylation induced by ATM is crucial for appropriate DDR activity.

### Role of RNF213 in cancer therapy

**Fig. 6:**
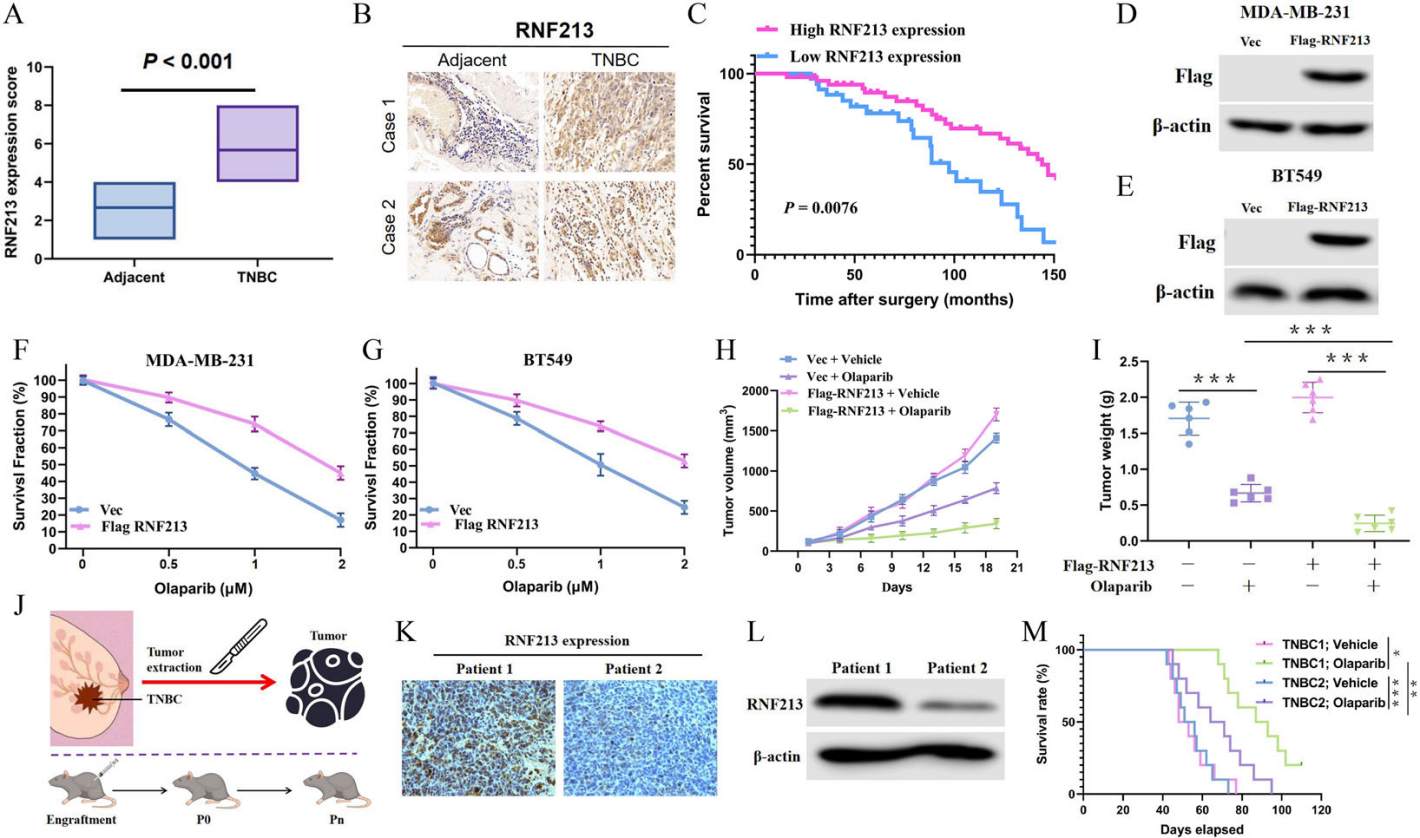
The role of RNF213 in response to cancer therapy. **A** IHC staining of RNF213 was evaluated by the German semi-quantitative scoring system dependent on the staining intensity. **B** Representative images of IHC analysis of RNF213 in the serial sections of tumor and paired adjacent normal tissues. **C** Kaplan-Meier estimates of overall survival of breast cancer patients with high and low RNF213 expression in a TMA. Log-rank test was used to compare the survival curves between groups. **D**–**G** Sensitivity of control and RNF213 over-expression MDA-MB-231 cells (**D**, **F**) and BT549 (**E**, **G**) cells to Olaparib was assessed by colony formation assay. **H**, **I** MDA-MB-231 cells stably expressing Vec or Flag-RNF213 were subcutaneously injected into nude mice. Tumor volume (**H**) and weight (**I**) were measured as indicated. **J** Schematic model for generating patient-derived tumor xenograft of TNBC. **K** Representative IHC micrographs showing RNF213 expression in the two patients with TNBC. **L** Immunoblot of RNF213 in the two TNBC PDX specimens. β-Actin was used as a loading control. **M** Growth curves of the TNBC-1 and TNBC-2 PDX models treated with vehicle or olaparib (n = 5 mice for each group).

RNF213 is upregulated in TNBC (Supplementary Fig. 3A–B), and increased expression of RNF213 is clearly associated with better prognosis in TNBC (Supplementary Figure 3C) but not in luminal breast cancer (Supplementary Fig. 3D) or Her-2-positive breast cancer (Supplementary Fig. 3E). The results of IHC analysis further indicated that RNF213 expression was significantly greater in TNBC tissues than in adjacent normal tissues (Fig. 6A, B, Supplementary Table 1), and TNBC patients with high RNF213 expression had a higher PARPi response rate and better outcomes than did those with low RNF213 expression (Fig. 6C and Supplementary Fig. 3F). Next, we overexpressed RNF19A in MDA-MB-231 and BT549 cells, which have relatively low RNF213 expression (Supplementary Fig. 3G), to examine whether RNF213 affects the response of TNBC cells to chemotherapy. The overexpression of RNF213 markedly sensitized MDA-MB-231 and BT549 cells to olaparib (Fig. 6D–G). MDAMB-231 cells were subsequently implanted subcutaneously to further confirm the role of RNF213 in the response to olaparib in vivo. RNF213 weakly promoted tumor growth in mice in the absence of olaparib treatment, but mice bearing tumors formed from RNF213-overexpressing MDA-MB-231 cells exhibited more marked tumor shrinkage in the olaparib treatment group (Fig. 6H, I). We also observed greater RNF213 expression in tumor tissues from olaparib-sensitive TNBC patients than in those from olaparib-resistant TNBC patients (Supplementary Fig. 3H). Moreover, fresh TNBC tumor tissues (TNBC-1 and TNBC-2) with different RNF213 expression levels were collected to establish PDX models (Fig. 6J–L). The PDX tumors derived from the TNBC-1 tissues, which had higher RNF213 expression than did the TNBC-2 tissues, displayed notably greater sensitivity to PARPi (olaparib) treatment than did the PDX tumors derived from the TNBC-2 tissues (Fig. 6M). These results indicate that high RNF213 expression renders TNBC cells hypersensitive to PARPi treatment.

## Discussion

TNBC, accounting for approximately 15% of all breast cancers and lacking progesterone receptor (PR) expression, estrogen receptor (ER) expression and human epidermal growth factor receptor (HER2) amplification, is one of the most aggressive and life-threatening types of human cancer [36–38]. The clinical prognosis of TNBC is typically dismal [39]. Inhibitors of PARP, which exhibit synthetic lethality with HR deficiency, have been approved for use in TNBC patients with mutations in BRCA1/2 or other HR deficiency-related factors. Clinical PARPi treatment markedly improved the outcomes of TNBC patients with BRCA1/2 deficiency. However, intrinsic and acquired resistance remain crucial issues.

In this study, we analyzed data from the TCGA database and a clinical sample microarray and reported that increased RNF213 expression is strongly associated with favorable survival outcomes in TNBC patients. RNF213 knockout mice were profoundly radioresistant, indicating that RNF213 is involved in the DDR. Furthermore, RNF213 inhibits HR and DNA end resection through RPA1 in a manner dependent on the ubiquitination of RPA1 via its catalytic E3 ligase activity. DUB activity is regulated by many mechanisms, including translational and posttranslational regulation, transcriptional modifications, and protein interactions [40, 41]. ATM is widely accepted as a key mediator in the DDR because of its ability to phosphorylate numerous factors containing closely spaced SQ/TQ motifs to modulate DDR cell cycle arrest and apoptosis [42–44], and various DUBs are activated through phosphorylation by ATM in response to DNA damage and modulate DNA damage repair [45–47]. For example, ATM phosphorylates SPOP at S119 and then facilitates HR repair over NHEJ during DNA replication by contributing to 53BP1 removal from chromatin [48]. The hyperphosphorylation of CtIP induced by ATM mediates the SUMOylation of CtIP at lysine 578 and then regulates DSB end resection [49]. We discovered that the phosphorylation of RNF213 at Ser217 by ATM increased the activity of RNF213 to promote RPA1 ubiquitination and inhibit HR repair and DNA end resection. ATM-dependent phosphorylation events are counteracted via PP2A-PR130 and other PP2A complexes [50]. On the other hand, the results of our in vitro and in vivo studies further indicated that high RNF213 expression results in greater PARPi sensitivity and better survival in TNBC patients.

RPA is indispensable for DNA recombination, repair, and replication [14, 51]. RPA is crucial for coating exposed ssDNA as protection from endogenous nucleases; additionally, RPA provides a platform for the recruitment of factors required for DNA repair and replication [52, 53]. RPA1 is the largest subunit of the RPA complex [54].

Moreover, the ubiquitination of RPA1 results in the loss of its ssDNA binding capacity [55, 56]. RPA1-depleted MDA-MB-231 cells were reported to have greater sensitivity to platinum and olaparib, a characteristic related to DSB accumulation [52]. Therefore, exploring the related regulatory molecular mechanism and identifying novel therapeutic targets are promising strategies for developing treatments for TNBC. Our results indicated that RNF213 sensitizes TNBC cells to PARPi treatment by mediating RPA1 ubiquitination.

Notably, DNA-targeted therapies, including PARPi treatment, can have both immunosuppressive and immunostimulatory effects, including regulation of the cGAS-STING signaling pathway [57, 58]. RNF213, which is induced by IFN-β, has been reported to promote the differentiation of regulatory T (Treg) cells and inhibit the development of autoimmune diseases [59]. Moreover, RNF213 was also identified as a modulator of IFN-γ–dependent pathogen restriction in human cells [60]. However, further exploration is needed to elucidate the effects of RNF213 combined with DNA-targeted therapies on the tumor immune microenvironment of TNBC.

**Fig. 7:**
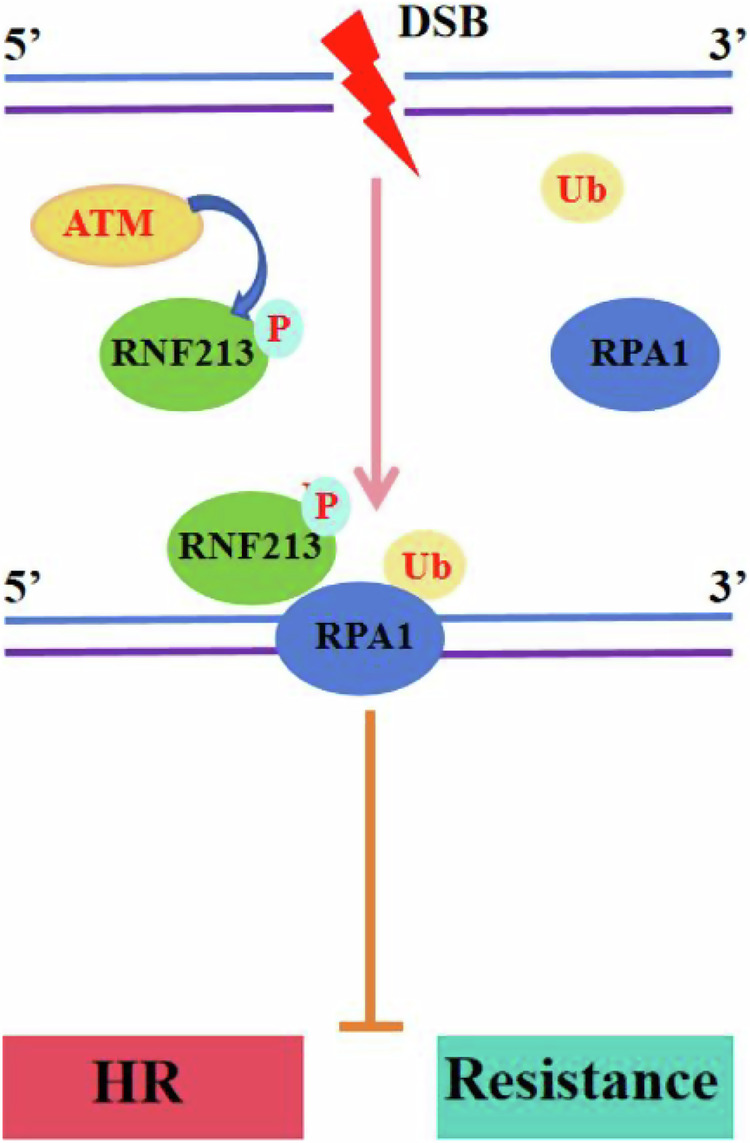
Working model of RPA1 regulation by RNF213.

Taken together, our findings suggest that RNF213, which is phosphorylated and thus activated by ATM following DNA damage, inhibits HR repair and DNA end resection through the modulation of RPA1 ubiquitination (Fig. 7). Moreover, RNF213 sensitizes TNBC cells to DNA-targeted therapy, and the RNF213 expression level might be a prognostic indicator in TNBC. Most importantly, our study revealed that targeting RNF213 in combination with PARP inhibitors represents a potential treatment option for patients with TNBC, and pharmacological modulators of RNF213 are currently under development. These findings might also be extended to other cancer types, such as ovarian cancer, pancreatic cancer, and prostate cancer. Therefore, future screening for RNF213 enzymatic inhibitors or pharmacological modulators that attenuate the DNA repair ability of RPA1 in cancer cells could enhance the effects of PARP inhibitors.

## Supplementary information

Supplementary materials

## Data Availability

All the data needed to evaluate the conclusions in the paper are presented in the paper and/or the Supplementary Materials. Additional data related to this paper may be requested from the authors.
