# Supplementary material for: Phosphorylation of RNF213 by ATM-mediated ubiquitination of RPA1 regulates homologous recombination repair and chemosensitivity
Source: Cell Death Dis. 2025 Oct 21;16(1):749. doi: 10.1038/s41419-025-08041-w (PMC12540995; doi:10.1038/s41419-025-08041-w)
Supplement: Supplementary file 1 — Supplementary materials [file 41419_2025_8041_MOESM1_ESM.docx]

**Supplementary Materials**

**Materials and methods**

**Cell culture**

HEK293T, U2OS, MDA-MB-231, and BT549 cells were purchased from ATCC. HEK293T, MDA-MB-231 were cultured in Dulbecco’s Modified Eagle Medium (DMEM) with 10% fetal bovine serum (FBS). U2OS and BT549 cells were cultured in McCoy’s 5 A and RPMI1640 with 10% FBS, respectively.

**Plasmids, reagents, and antibodies**

CHX, IgG agarose, anti-Flag agarose, 3×Flag peptide and ATM inhibitor KU55933 were purchased from Sigma Aldrich. Olaparib was purchased from Toronto Research Chemicals. Anti-RNF213 (PA5-51902, 1:1000) was purchased from Thermo Fisher; anti-RPA32 (sc-56770, 1:2000), anti-Ub (sc-8017, 1:2000), anti-BRCA1 (sc-6954 1:1000), anti-CtIP(sc-271339, 1:1000) and anti-GFP (sc-9996, 1:1000) were purchased from Santa Cruz; anti-53BP1 (NB100-304, 1:1000) was from Novus Biologicals; anti-Flag (F1804, 1:2000) and anti-β-actin (A2228, 1:3000) were purchased from Sigma; anti-pS345 Chk1 (2348, 1:1000), anti-Myc Tag (2276, 1:2000) and anti-SQ/TQ motif (9607, 1:1000) were purchased from CST; anti-RAD51 (GTX100469, 1:1000) was purchased from Genetex. Anti-γ-H2AX (05-636, IF-1:1000) and anti-MDC1 (05-1572, IF-1:1000) were purchased from Millipore.

**shRNA transfection**

The shRNA-mediated knockdown was utilized to stably silent RNF213 gene expression. GFP(Puro)-lentiviral particles carrying RNF213 shRNA and negative control were purchased from Tsingke Biotechnology Company (BJ, CHN). Once cells had grown to 70-80% confluence in a six-well plate, they were infected with lentiviral particles for 24 h and cultured in refreshed medium for another 48 h. Then, 2 µg/mL puromycin (Sigma, P8833, US) was used to screen positive cells. The sequence

of the shRNAS was as follows: human RNF213 shRNA1, 5’-CAAUGUCGACUUUGAUAAACU-3’; shRNA2, 5’-CGTCCAATTTACGTCACCGCGTCAT-3’.

human RPA1 shRNA1, 5’-CCCTAGAACTGGTTGACGAAA-3’; shRNA2, 5’-CCGCATGATCCTGTCAGTAAATAT-3’.

human 53BP1 siRNA1, 5’-GAGCUGGGAAGUAUAAAUU-3’; shRNA2, 5’-GGACUCCAGUGUUGUCAUU-3’.

**Cell counting kit-8 (CCK8) assay**

For CCK8 assay, 2000 cells were seeded per well in a 96-well plate and incubated overnight at 37 ℃. Thereafter, the plate was placed in a three-gas incubator with 1% O_2_. For CCK8 detection, a 10% CCK8 working solution (MCE; Cat# HY-K0301) was

added to the wells and the plate was incubated for 2 h in the dark at 37 ℃. Absorbance was read at 450 nm using a microplate reader (BioTek, USA). The assay was repeated three times for each sample.

**Colony formation**

Totally, 8000 cells were plated in each well of 6-well plates and then treated with indicated treatments. After incubated for about 14 days at 37 °C, colonies were stained with 5% GIEMSA and counted.

**Cell cycle analysis**

Cells were seeded into 6-well plates (5 × 10^5^ cells/well). After 72 h, the cells were collected and fixed with 70 % ethanol at 4 °C overnight. Then cells were treated with RNase A and propidium iodide for 30 min at 37 °C and analyzed by flow cytometry.

**Western blot and immunoprecipitation**

Cells were harvested and lysed with NETN buffer (20 mM Tris-HCl, pH 8.0, 100 mM NaCl, 1 mM EDTA, 0.5% Nonidet P-40 with 50 mM 10 mM NaF, and 1 mg per ml each of pepstatin A and aprotinin. After centrifugation at 12,000 × g for 15 min, supernatant containing proteins was immunoprecipitated by incubating indicated antibodies or agarose beads overnight at 4 °C. The immunoprecipitates were washed with NETN and then centrifuged at 800 × g for 1 min for three times. The immunoprecipitates were added with 50 μL 1× Laemmli buffer and then boiled for sodium dodecyl sulfate polyacrylamide gel electrophoresis separation, thereafter detected with indicated antibodies. All of uncropped blots are available in source data file.

**Denaturing Ni-NTA pull-down**

Cells were harvested and lysed in buffer composed of 8 M Urea, 0.1 M NaH2PO4, 30 mM NaCl and 0.01 M Tris (pH 8.0). Lysates were then sonicated to shear DNA and incubated with Ni-NTA agarose beads for 2 h at room temperature. After washing the beads with urea wash buffer (8 M Urea, 0.1 M NaH2PO4, 300 mM NaCl and 0.01 M Tris (pH 8.0)) for 5 times, the immunocomplexes were added with 1× Laemmli buffer and subjected to western blot.

**In vitro deubiquitination assays**

HEK293T cells were transfected with both HisUb and RPA1, then ubiquitinated RPA1 was purified using Ni-NTA agarose beads and incubated with purified FLAG–RNF213 in deubiquitination buffer (50 mM Tris-HCl pH 8.0, 50 mM NaCl, 1 mM EDTA, 10 mM DTT, 5% glycerol) at room temperature for 4 h.

**Immunofluorescence staining**

U2OS cells were cultured on coverslips for 24 h before experiments. For γ-H2AX, MDC1,53BP1, and BRCA1 foci, cells were fixed with 4 % paraformaldehyde (PFA), permeabilized with 0.5% Triton X-100; For RAD51 foci, cells were permeabilized with 0.5% Triton X-100 on ice for 5 min, then fixed with 4% PFA; Cells were fixed and permeabilized with methanol: acetone (1:1) at −20 °C for 20 min to detect RPA32 foci. Following this, coverslips were washed with phosphate-buffered saline (PBS) 3 times, cells were blocked with 5% goat serum for 30 min and then incubated with primary antibodies (4 °C overnight). After washing with PBS, coverslips subsequently incubated with corresponding Alexa Fluor 488 or 594-conjugated secondary antibodies for 1 h at room temperature. and the nuclei were stained with 4'6-diamidino-2-phenylindole (DAPI). The coverslips were mounted onto glass slides with anti-fade solution and visualized by a Nikon ECLIPSE E800 fluorescence microscope. Foci intensity was quantified using Image J.

**HR assay**

Cells expressing indicated shRNAs or constructs were transfected with DR-GFP, pCBA-I-SceI, and pCherry. After 2 days cells were harvested and analyzed by fluorescence-activated flow cytometry (FACS) to examine the percentage of GFP-positive cells. Results were normalized to control group.

**Supplementary Figures**

**Supplementary Figure 1**


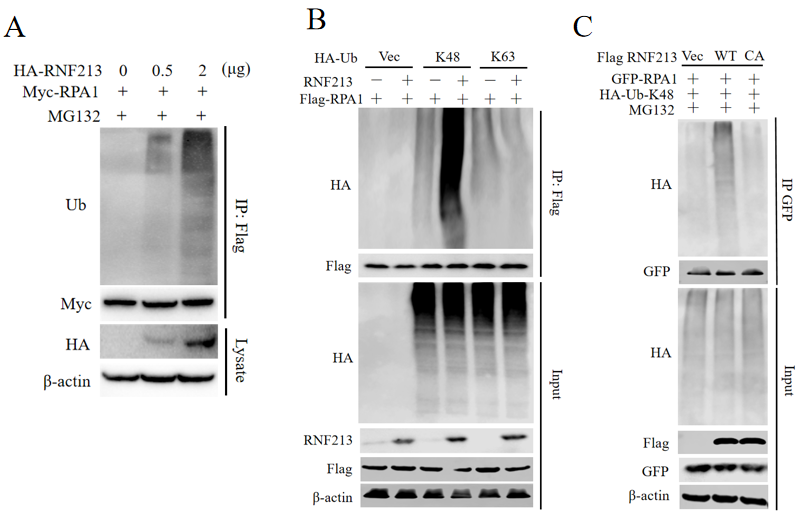


**Supplementary Figure 2**


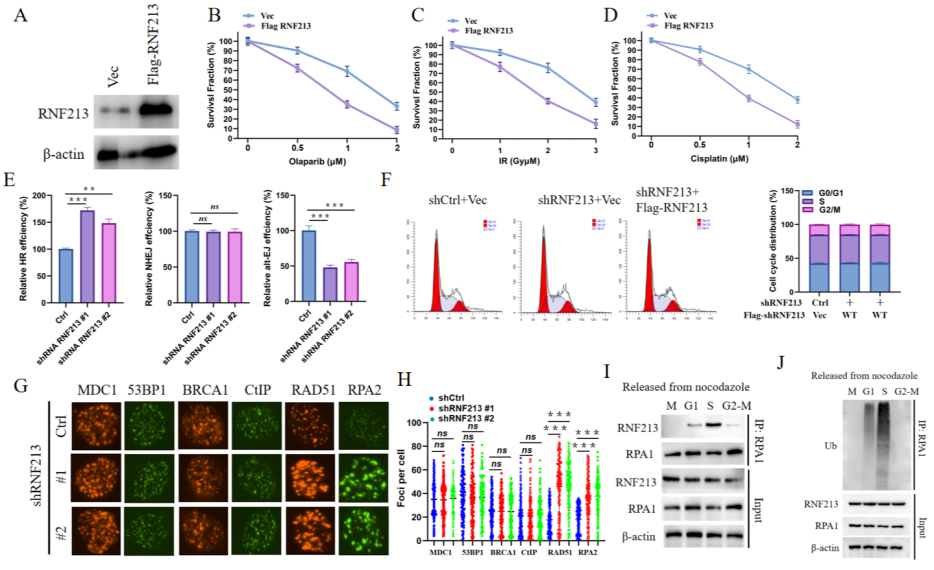


**Supplementary Figure 3**


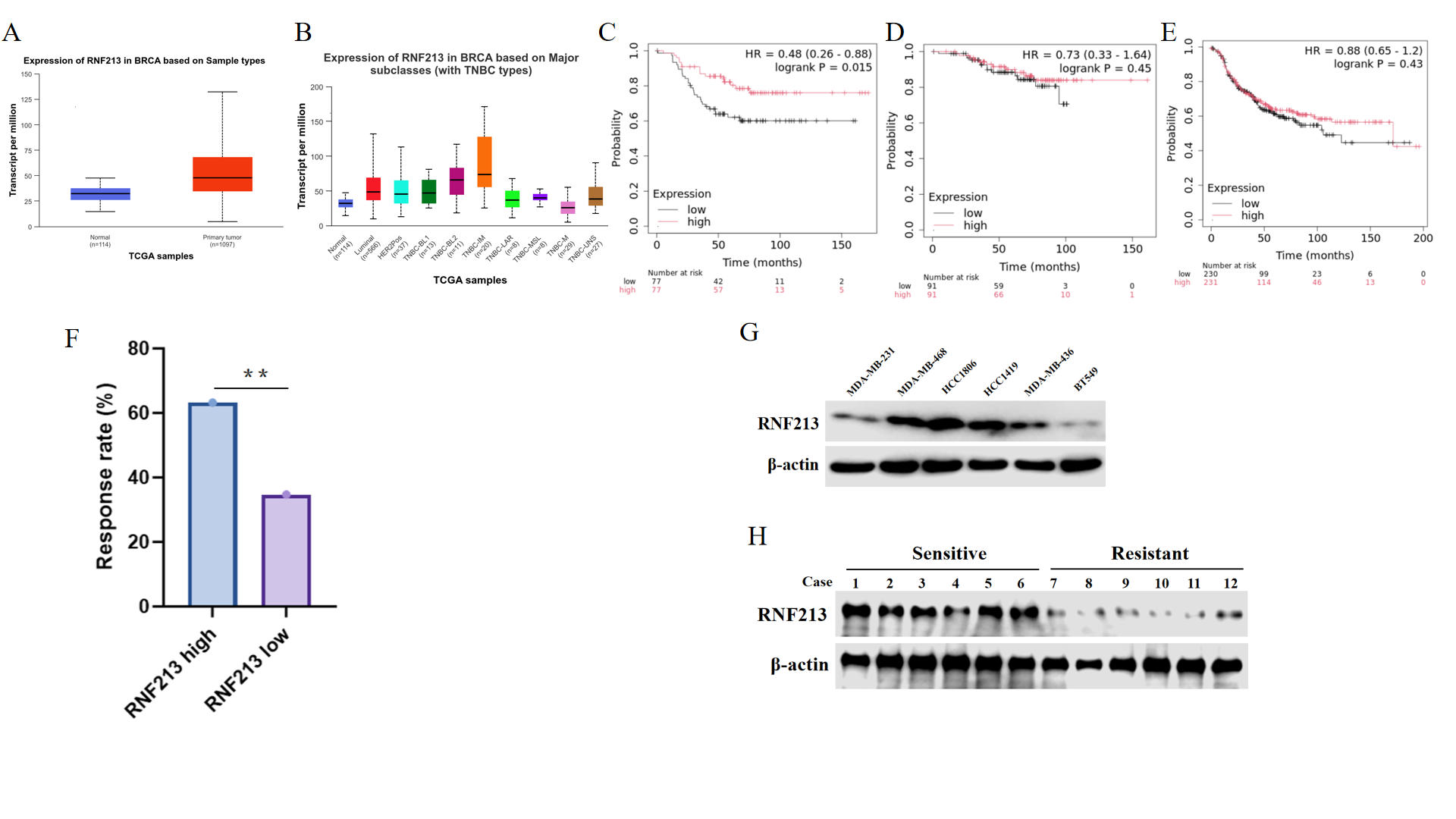


**Supplementary Figure Legends**

**Supplementary Figure 1. RNF213 ubiquitinated RPA1 via K48-linked polyubiquitin chains**

A. HEK293T cells were transiently transfected with indicated HA tagged ubiquitin and Myc-tagged RPA1. After 48 h, cells were treated with MG132 (50 μM) for 3 h before collecting. Cell lysates were subjected to immunoprecipitation with anti-Myc agarose beads, and then blotted with the indicated antibodies.

B. HEK293T cells stably expressing control or RNF213 shRNAs were transiently transfected with indicated HA tagged ubiquitin and Flag-tagged RPA1. After 48 h, cells were treated with MG132 (50 μM) for 3 h before collecting. Cell lysates were subjected to immunoprecipitation with anti-Flag agarose beads, and then blotted with the indicated antibodies.

C. HEK293T cells expressed RNF213 WT or CA were transiently transfected with indicated HA-K48 lysine-specific mutant constructs and Flag-tagged RPA1. After 48 h, cells were treated with MG132 (50 μM) for 3 h before collecting. Cell lysates were

subjected to immunoprecipitation with anti-Flag agarose beads, and then blotted with the indicated antibodies.

**Supplementary Figure 2. RNF213 inhibits HR and increases sensitivity to DNA-damaging agents.**

A. U2OS cells stably expressing Vector or Flag-RNF213, the blots were probed with indicated antibodies.

B-D. The sensitivity of Vector and Flag-RNF213 expressed U2OS cells to Olaparib (B), IR (C) and cisplatin (D) was assessed by colony formation assay. Error bars are means ± s.d. of three independent experiments.

E. Control or RNF213-depleted HEK293T cells were transfected with an HR or NHEJ or alt-EJ reporter for 48 h. Cells were then harvested for HR or NHEJ analysis. Error bars represent means ± s.d. of three independent experiments.

F. Cell cycle was analyzed by flow cytometry in control (Ctrl) and RNF213 knockdown U2OS cells stably expressing Vec or Flag-RNF213. Error bars

represent means ± s.d. of three independent experiments.

G-H. Control or RNF213 knockdown U2OS cells were treated with IR (3 Gy, 1 h for MDC1, 53BP1, BRCA1; 3 Gy, 4 h for RAD51 and RPA32), and indicated foci were detected by immunofluorescence. Nuclei were visualized with DAPI (blue). Representative images are shown G. Quantification of focus signals per cell (each dot represents a single cell, n = 100) is shown in H. Error bars represent means ± s.d. of three independent experiments.

I. MDA-MB-231 cells were synchronized with nocodazole (100 ng/ml) for 12 hours and released into the cell cycle. At the indicated time points, cells were harvested for cell cycle and then co-IP and WB analysis with the indicated antibodies.

J. MDA-MB-231 cells were transfected with HA-Ub-K48 construct and released from nocodazole. Cells were harvested for IP at indicated time points as I.

**Supplementary Figure 3. The role of RNF213 in response to cancer therapy.**

A-B. RNF213 expression in breast cancer in TCGA

C-E. Kaplan-Meier poverall survival curves of patients with TNBC (C), luminal breast cancer (D) and Her-2-positive breast cancer (E).

F. The response rate to Olaparib of TNBC patients with different RNF213 expression.

G. Expression of RNF213 in human TNBC cell lines.

H. RNF213 expression in tumor tissues of Olaparib sensitive and resistant TNBC patients.

Supplementary Table 1

Correlation between clinical feature and RNF213 expression in TNBC

| Characteristics | Groups | N^2^ | RNF213 expression | | *P* value |
| --- | --- | --- | --- | --- | --- |
|  |  |  | High | Low |  |
| Ages | <60 | 49 | 29 | 20 | 0.614 |
|  | ≥60 | 37 | 22 | 15 |  |
| Histological grades | Ⅰ-Ⅱ | 43 | 23 | 20 | 0.027 |
|  | Ⅲ | 43 | 28 | 15 |  |
| Tumor size (cm) | <3 | 36 | 21 | 15 | 0.366 |
|  | ≥3 | 50 | 30 | 20 |  |
| Lymph node metastasis | NO | 41 | 24 | 17 | 0.243 |
|  | YES | 45 | 27 | 18 |  |
| Clinical stage | Ⅰ-Ⅱ | 59 | 35 | 24 | 0.196 |
|  | Ⅲ-Ⅳ | 27 | 16 | 11 |  |
